# Digital platform-based conceptual framework for food environment research in China

**DOI:** 10.1017/S1368980024002209

**Published:** 2025-03-13

**Authors:** Na Cong, Keumseok Koh, Mei-Po Kwan, Hongsheng Zhang

**Affiliations:** 1 Department of Geography, Faculty of Social Science, The University of Hong Kong, Hong Kong, Hong Kong Special Administrative Region of China; 2 Department of Geography and Resource Management, The Chinese University of Hong Kong, Hong Kong, Hong Kong Special Administrative Region of China; 3 Institute of Space and Earth Information Science, The Chinese University of Hong Kong, Hong Kong, Hong Kong Special Administrative Region of China

**Keywords:** Food environment, Digital platform, Obesity, China, Systematic framework

## Abstract

China has dedicated significant efforts to preventing obesity, but the rising prevalence of overweight and obesity remains a pressing public health issue. Therefore, unique solutions are required to address this challenge in China. As a research priority, the food environment plays a pivotal role in addressing overweight and obesity. However, research on this topic in China lags behind that in other developed countries, and the conflicting global evidence on the association between the food environment and obesity cannot be directly applied to policymaking and intervention in China. In addition, the rapid advancement of digital technology has introduced complexities and uncertainties in the food environment. To address these challenges, we propose an alternative research framework through (a) dissecting the challenges associated with defining and measuring the food environment, (b) reorganising the relationship chains between the food environment and human diet/health and (c) taking into consideration digital platforms as crucial monitoring tools for studying the food environment. Our framework aims to unlock the potential of food environment research in the digital age, ultimately striving to tackle the overweight and obesity issues in China.

Obesity has emerged as a critical public health concern in China, with over half of the adult population classified as overweight or obese^(1)^, placing China at the forefront of the global obesity epidemic^(2)^. Research indicates that obesity is influenced by a complex interplay of factors including obesogenic environments and societal dynamics. In particular, the food environment plays a pivotal role in preventing obesity^(3,4)^. However, despite the introduction and investigation of the food environment in China^(5)^, a comprehensive examination remains lacking^(6)^. Food outlets offering diverse food options are deeply integrated into our daily lives, interacting with transportation, economic and urban systems to impact health. Insights from international studies may not be fully applicable to China’s distinct social patterns and varied environments. A deeper understanding of China’s food environment requires an analysis within both national and global contexts.

Furthermore, the rapid growth of digital technologies and innovative business models in China has significantly transformed societal interactions with the food environment over the past two decades^(4,7)^. Although the challenges and health implications of the food delivery industry have been noted^(8)^, strategies for leveraging these platforms to improve diet quality and health are unclear. Amid the complexity of the food environment and its dynamic interactions with broader systems, particularly in the face of technological advancements and societal shifts in China, a comprehensive understanding of the food environment is vital for obesity mitigation^(9)^. This commentary explores the food environment within a broader context, addresses key research issues and develops a framework suitable for the Chinese context.

## The research map of the food environment

**Figure 1.**
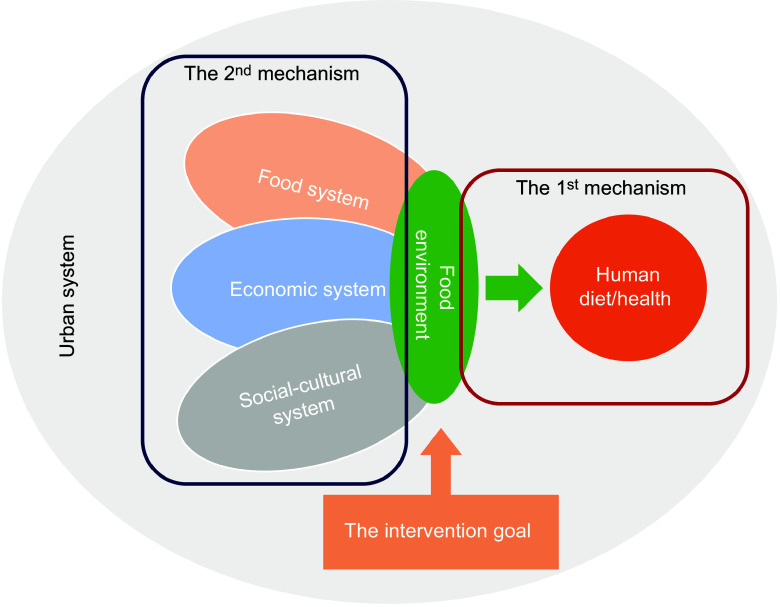
Food environment research map according to the gaps summarised by the High-Level Panel of Experts report (2017).

In 2017, the UN High-Level Panel of Experts on Food Security and Nutrition identified three key research gaps in the study of the food environment (Fig. 1)^(4)^. The first gap concerns the effect of the food environment on human health (the 1^st^ mechanism). The second gap explores how external factors, such as transportation, land use and sociocultural environments, influence the food environment (the 2^nd^ mechanism). The third gap focuses on developing a comprehensive theoretical framework to measure and understand the dynamics of the food environment for future intervention.

Previous decades of research have focused on the health effects (the 1^st^ mechanism), but inconsistent findings have hindered the development of obesity mitigation strategies^(10–15)^. However, understanding the 1^st^ mechanism does not represent the entirety of research on food environments. The food environment is linked to various urban subsystems and other systems and is constrained by them. For example, transportation issues can impede food distribution and urban planning can indirectly shape food environments. To generate meaningful findings for human health policies, it is essential to address these gaps comprehensively in a broader context. This involves clarifying its external links with multiple urban subsystems (the 2^nd^ mechanism), internal differences and connections with other food system components, and defining the food environment more precisely (the intervention goal). The most critical research gap lies in the development of a theoretical framework that accurately defines and measures the food environment, which is crucial for uncovering the two mechanisms. Therefore, our priority lies in thoroughly examining and dissecting the challenges in defining and measuring the food environment to develop a conceptual framework tailored to the Chinese context.

## Deconstructing the traditional definition of food environment: identifying and highlighting key elements

Defining the food environment and its key components poses a great challenge^(3)^. Existing definitions, which integrate physical, economic, political and sociocultural contexts and various influencing factors, have not effectively established a link between the food environment and population health outcomes. This is because these contexts not only influence the food environment but also directly or indirectly affect population health through other pathways. The complexity involved makes it exceptionally challenging to explain the relationship between food environment and population health. Therefore, the definition of food environment should identify its distinctive characteristics and specify the specific pathways through which it affects population health.

Tracing back to the inception of food environment research may shed light on this question. The primary focus of these studies was initially to address the rising obesity epidemic, as the overabundance of food provided by the food environment is the main driver of this global health concern^(16)^. Additionally, the dietary risk factors identified in studies on the global burden of disease are all based on evidence of the nutritional composition of food^(17)^. Thus, food and nutrition, but not other attributes such as food quality and safety, are the core components of the food environment^(18)^. In contemporary society, the accessibility of food is not ubiquitous but rather concentrated in specific locations. Spatial proximity between food and consumers plays a pivotal role in determining the dynamics of the food environment and its interaction with individuals. Furthermore, the spatial distribution of food plays a fundamental role in establishing evidence regarding factors that influence the food environment. Therefore, the spatial location of food is the second key component of the food environment.

When considering the quantity or other characteristics of food as attributes of the food environment, it is essential to recognise that the physical, economic, political and sociocultural contexts outlined in the High-Level Panel of Experts definition should be regarded as impact factors that contribute to changes in the food environment, rather than constituting the food environment itself. To this extent, we identified the fundamental components that constitute the food environment as well as the external systemic factors that influence it.

A noticeable issue arising from the vague definition of traditional food environments is the proliferation of various terms derived from it. Different studies refer to the food environment using terms such as market food environment^(19)^, retail food environment^(11,20)^, community/neighbourhood food environment^(21)^, public food environment^(18,22)^, local food environment^(10,18)^ and workplace food environment^(23)^. However, the distinctions and connections between these terms are overlooked.

## Measurement issues

Measuring the food environment in multiple dimensions may address several challenges.

First, primary measurements in community-scale studies often target specific outlets, such as supermarkets^(24,25)^ and restaurants^(26–28)^, or specific settings, such as residential areas or schools^(29)^. In some cases, the availability of certain types of food has been assessed^(30,31)^. On a broader scale, food environments have often been measured by counting the number of different food outlets in many studies^(23,32–34)^. However, the reliability of these methods has been questioned^(18,35–37)^. Given that dietary risk factors identified by the global burden of disease research are universally applicable^(38)^, it may be more effective to develop scale-independent measurement methods based on these factors as demonstrated by a recent study^(18)^.

Second, the incorporation of the digital dimension into the food environment framework has been under-discussed. It remains unclear whether the digital food environment (DFE) requires a separate measurement method or whether it can be integrated into the overall framework. Although a recent study touched on digital settings and culture, the theoretical framework for comprehending the DFE and its integration into the overall framework is in its infancy^(39)^. Simply adding the digital dimension to the existing measurement dimensions of the food environment will only complicate measurements, without fundamentally solving the problem.

Third, the lack of transparency regarding the development of measurements and extraction of information on the different dimensions of food environments from their data should be addressed^(35)^. One review found that only half of the studies followed rigorous systematic steps, hindering the comparability and replicability of research findings^(40)^. A protocol to standardise food environment measurements within the overall framework is needed to ensure an effective comparison of results across contexts and foster a more comprehensive understanding of the impacts of food environments on health and sustainability.

## A framework for food environment research

**Figure 2.**
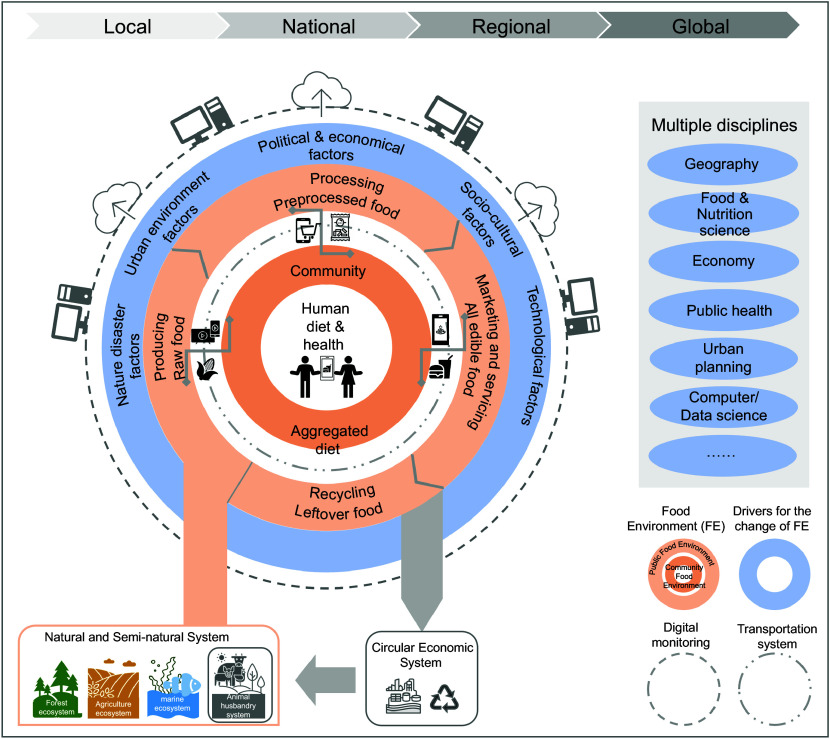
A conceptual framework for diet, food, food environment, food system and broader systems in the digital age.

In light of the above discussions, we characterise the food environment as a specialised environment, encompassing food items and their nutritional value, as well as the physical locations where they are available^(18)^. As the core of the food system, it bridges the extensive Earth system and persistently interacts with the relevant population via technological, economic, social and cultural factors, ultimately influencing human health. Accordingly, we propose a conceptual framework as depicted in Fig. 2.

The traditional food supply chain involves several steps, from harvesting food to making it available to consumers at retail and service stages, including production, storage, distribution, transportation, processing, packaging and retailing^(4)^. However, the digital age has brought significant changes, providing consumers with unprecedented access to food products. Digital platforms for food sales and services, such as online delivery, e-commerce, live streaming and modernised delivery systems, have broadened consumer access to diverse food choices. These changes have broken down the traditional barriers and allowed people to connect directly with producers, processors, retailers and distributors. The resulting public food environment encompasses various foods and their suppliers throughout the food supply chain^(22)^, including all market and retail food environments.

Individual food items are aggregated into dishes or meals during food services or cooking at home, ultimately forming part of the consumer’s diet. The goal of both the retail and food service sectors is to deliver food to the consumer’s location for consumption. This food, delivered to communities, represents the aggregated diet of people in those places and constructs the community food environment. The community food environment, as defined here, includes the neighbourhood food environment, local food environment, workplace food environment and other community areas where people receive food. The community food environment plays a crucial role in exploring the association between individual dietary choices and the public food environment. A thorough understanding of the public food environment is essential for formulating effective macro-intervention policies, whereas a comprehensive understanding of the community food environment is valuable for implementing practical intervention practices.

The food environment is influenced by various factors, including natural disasters, urbanisation, political and economic shocks, sociocultural norms and technological advancements, all of which also impact human diet and health. The complexity of these factors requires a multidisciplinary approach involving fields such as geography, food and nutrition science, economics, public health, urban planning and computer and data science. By combining these diverse disciplines, we can gain a comprehensive understanding of the factors influencing the food environment and develop effective intervention strategies and urban planning approaches to address obesity and promote healthier food environments.

Digital technologies have infiltrated nearly every facet of human existence, including the food environment and its contributing factors. These technologies serve as crucial tools for monitoring and analysing the food environment and its impact factors. However, it is important to note that the foundational definition of the food environment currently in use does not differentiate between the physical food environment^(41)^ and the DFE^(39)^. This is because food purchases frequently originate from physical food outlets, even though digital technologies have significantly transformed the way individuals engage in food environments. Essentially, integrating digital aspects into the food environment for DFE does not change the core relationship between the food environment and human diet/health because the digital dimension of the food environment cannot exist independently of the physical food environment. In our framework, the digital aspects are identified as belonging to the technological factor domain. The current adoption of terms such as DFE not only directs researchers’ attention to the digital aspects of the food environment but also emphasises the crucial role of digital technology in facilitating comprehensive monitoring of the food environment. This has the potential to catalyse a ‘data revolution’ in food environment research, ultimately leading to a paradigm shift in the field^(4)^.

In addition, although the transportation system serves as the key means of delivering food from providers to consumers, it does not influence the core relationship between the food environment and human diet/health, which is similar to digital technology. Thus, the transportation system was not highlighted in our framework.

Our framework provides several benefits. At the micro level, integrating nutrition into food environment definitions enhances measurement approaches informed by global disease burden insights. This can lead to effective and interpretable measurements that may be incorporated into the Food Systems Countdown Initiative^(42)^. At the macro level, the framework enables food environment research to extend beyond merely addressing the global obesity epidemic from a nutritional or epidemiological perspective and serves as a critical tool in addressing the global syndemic (including obesity, undernutrition and climate change)^(43)^ and other environmental issues. Furthermore, it broadens the scope of Earth system modelling to include human social systems^(44)^, given that the food environment is a central link among various societal systems (as shown in Fig. 1), impacting both human and planetary health.

## Conclusions

China’s obesity issue is intertwined with the global challenges of obesity and malnutrition. The slow progress in addressing obesity and other forms of malnutrition^(45)^, despite significant efforts, indicates that global malnutrition is a complex challenge. China’s rapidly evolving social environment has further complicated the task of addressing this complex challenge. To tackle these intricate issues, an integrated approach driven by evidence-based research within an effective framework is necessary^(43,46,47)^. Rethinking the food environment in the digital age allowed us to identify new opportunities to address these issues. Clarifying the foundational concepts of the food environment and its interconnections with various systems is pivotal for unravelling critical solution pathways. The framework proposed in this paper offers a fresh perspective on addressing these challenges. However, to truly unlock the health-promoting potential of the food environment and achieve a win-win situation for both human and planetary health, fostering interdisciplinary and cross-regional collaboration is essential.
